# An experimental study: the effect of *S*. *boulardii* on abemaciclib-induced diarrhea

**DOI:** 10.55730/1300-0144.5557

**Published:** 2022-07-24

**Authors:** İbrahim Ethem CAKCAK, Yusuf Emre AYTİN, Sezin SAYIN, Ahmet KÜÇÜKARDA, Ali GÖKYER, İvo GÖKMEN, Erkan ÖZCAN, Selçuk KORKMAZ, Ebru TAŞTEKİN, İrfan ÇİÇİN

**Affiliations:** 1 Department of General Surgery, Faculty of Medicine, Trakya University, Edirne, Turkey; 2 Faculty of Medicine, Trakya University, Edirne, Turkey; 3 Department of Medical Oncology, Faculty of Medicine, Trakya University, Edirne, Turkey; 4 Department of Biostatistics and Medical Informatics, Faculty of Medicine, Trakya University, Edirne, Turkey; 5 Department of Pathology, Faculty of Medicine, Trakya University, Edirne, Turkey

**Keywords:** Breast cancer, abemaciclib, *Saccharomyces boulardii*, diarrhea

## Abstract

**Background/aim:**

In our study, we aimed to investigate the protective effects of *Saccharomyces boulardii* on abemaciclib-induced diarrhea model, which is a commonly used drug in breast cancer.

**Materials and methods:**

Thirty rats were divided into 3 groups as control (Group 1), abemaciclib (Group 2), and abemaciclib + *Saccharomyces boulardii* (Group 3) groups. The clinical status, body weight, and defecation status were monitored daily. At the end of the 15-day experiment period, the rats were killed with high-dose anesthesia and the resected small intestine segments were evaluated histopathologically. Lesions were classified according to thickening of the villus, inflammation and edema of mucosa and intraepithelial leukocyte accumulation. Then, mean values of both crypt depths and villi thicknesses were calculated for each rat. Normal distribution assumption was controlled with the Shapiro-Wilk test. One-way analysis of variance for normally distributed variables in the comparisons of more than two independent groups and Kruskal-Wallis test for nonnormally distributed variables were used. The significance value was accepted as 0.05.

**Results:**

There was one death in Group 3, but none in the others. There were no findings of mucositis in Group I. There was mild diarrhea and weight loss in only one rat in Group 1. For the comparison of the severity of diarrhea (72.5%/39%) and weight loss (72.5%/45%), a decrease was found in Group 3 according to Group 2 (p < 0.01). Histopathological findings such as edema, inflammation, and intraepithelial leukocyte accumulation also showed a decrease in Group 3 compared to Group 2 (p < 0.01).

**Conclusion:**

*Saccharomyces boulardii* should be considered as a treatment option in abaemaciclib (chemotherapy)-induced diarrhea. Further comparative studies and in vivo human randomized controlled studies can be conducted in the future.

## 1. Introduction

Breast cancer is the most common cancer in women, constituting 30% of cancers diagnosed each year, excluding skin cancers, and it is the second most common cause of cancer-related death in women [1,2]. Endocrine therapies provide a better prognosis in hormone-receptor positive (HR+) breast cancer. Cycline dependent kinase (CDK) 4/6 inhibitors help break the resistance against endocrine therapy and improve the outcomes of endocrine therapy in breast cancer. In recent years, CDK 4/6 inhibitors palbociclib, ribociclib, and abemaciclib have been shown to improve survival in metastatic breast cancer [3–5]. In addition, abemaciclib was found to increase invasive disease-free survival (IDFS) in an adjuvant study [6].

Cyclin-dependent kinases 4 and 6 (CDK4 and CDK6), which are critical regulators of cell cycle progression, have important effects on breast carcinogenesis and endocrine therapy resistance [7, 8]. Diarrhea is one of the most common side effects of abemaciclib, which can be used in adjuvant and metastatic disease treatment. In MONARCH 1–3 studies, all grades of diarrhea (grade 1 to 4) were reported with a rate of 90.2% (MONARCH 1), 86.4% (MONARCH 2), and 81.3% (MONARCH 3) [5–10]. In the monarchE study, which is the adjuvant study, the frequency of grade 1–4 diarrhea was determined as 82,2% **[6].**

The clinical efficacy of abemaciclib, a CDK4 and CDK6 inhibitor, was first demonstrated by the MONARCH 1 study [5]. While endocrine therapy was first applied to patients with advanced breast cancer who are hormone receptor (HR) positive and Human Epidermal Growth Factor Receptor (HER) negative, abemaciclib has been shown to be an effective monotherapy agent in this study [5]. Also, according to the study by Goetz et al., the combination of abemaciclib and a nonsteroidal aromatase inhibitor get significantly better progression-free survival and objective response rate, making it an effective agent in the treatment of HR+, HER2− advanced breast cancer [10].

In the subgroup analysis of Takahashi et al.’s MONARCH 3 study, 82.3% of the patients developed any grade diarrhea and 9.5% had grade III or higher grade diarrhea, 13.8% of the patients who developed diarrhea due to abemaciclib required dose reduction and 15.3% dose omission [11].

*Saccharomyces boulardii* (*S. boulardii*) is a nonpathogenic yeast and has been demonstrated to be used in the treatment of diarrhea [12,13]. In the study conducted by Duman et al., the effects of saccharomyces on the side effects caused by clarithromycin and methotrexate treatment on rats were investigated [14]. In this study, although Saccharomyces had no effect on intestinal motility, they showed that it reduced lipid peroxidation, glutathione level, myeloperoxidase activity. In histological examination, they showed that it acts by reducing crypt and surface epithelial degeneration, villus atrophy, and inflammatory cell infiltration.

Loperamide is used in the management of chemotherapy-induced diarrhea since it was used in the MONARCH 2 study, while there is no proven positive effect yet [15]. Our study aims to investigate whether a treatment method other than loperamide, a probiotic agent, *S. boulardii*, can also be effective in diarrhea associated with malignancy treatment, CDK4 and CDK6 inhibitors are used in this study.

We aimed to create a diarrhea model due to abemaciclib and investigate the protective effect of *S. boulardii* in the diarrhea model. In line with the results of this study, we also aimed to show that *S. boulardii* can also be among one of treatment options in chemotherapy-induced diarrhea.

## 2. Materials and methods

The study was reviewed and approved by the Institutional Animal Care Committee. *S. boulardii* (Reflor Sase, Sanofi Synthelabo, France) and Abemaciclib (Campto, Aventis Pharma Ltd. Dagenham Essex, England) used in the study were purchased commercially.

Animal experiments were carried out in the Experimental Animals Laboratory of Trakya University, accompanied by a veterinarian, between January 12, 2022 and January 30, 2022. Subsequently, pathological examinations were made and reported.

Approximately 256 ± 30 g weighting male Sprague-Dawley rats were used in this experiment. Considering that hormonal activities in female rats may have an effect on the model to be created, it was decided to use male rats. The temperature of animal room was maintained at 22 ± 2 °C with a 12-h light-dark cycle. Acclimatization was maintained with tap water throughout the experimental periods. There were one control group (Group 1) and two study groups (Group 2 and Group 3) and each group had 10 rats determined by randomization.

No treatment was applied to the rats in the control group. Rats in Group 2 were administered abemaciclib (120 mg/kg) alone orally in methylcellulose solution once a day for fifteen consecutive days (days 1–15) via an orogastric feeding tube. In addition to the 15-day abemaciclib treatment, the rats in Group 3 were given *S. boulardii* (800 mg/kg) with an orogastric tube for a total of 18 days, 3 days before the experiment and 15 days during the experiment.

The general clinical status, body weight, and defecation status of the rats were monitored daily. The diarrhea was scored in 4 stages according to severity. In this classification, the normal stool was termed “normal”, slightly wet stool with no staining of the feathers in the perianal region was termed “mild”, watery, unformed stool with moderate staining of the feathers in the perianal region was termed “moderate”, and completely watery stool with severe staining of the feathers around the anus was termed “severe” [16].

Rats were killed with high-dose anesthesia on the 15th day of the experiment. Tissue samples of the proximal small intestine of all rats were fixed in 10% buffered formaldehyde for 12 h and graded alcohols were used for the procedure. Sections of 5 μm thickness were taken from the paraffin-embedded tissues and stained with hematoxylin and eosin. A light microscope (Nikon Eclipse E600, Japan) was used for the examination of tissue slides and lesions were graded from 0 to 3. According to this classification, no lesion was graded as “0”, a mild lesion as “1”, a moderate lesion as “2”, and a severe lesion as “3”. Lesions were classified according to thickening of villus, inflammation of mucosa, edema of mucosa and intraepithelial leukocyte accumulation [17]. A computer using telepathology image analysis software connected to a Zeiss MC 80 DX Axioplan2 imaging microscope (Zeiss digital microscope, Axioplan 2 imaging The Universal Microscope System, Germany) was used for the evaluation of intestinal mucosa of rats. Measurements were made for the deepest three crypts and thickest three villi in each rat. Then, mean values of both crypt depths and villi thicknesses were calculated for each rat for statistical analysis. One pathologist (E.T.) performed the pathological examination twice.

### 2.1. Statistical analysis

Normal distribution assumption was controlled with the Shapiro-Wilk test. One-way analysis of variance was used for normally distributed variables in the comparisons of more than two independent groups. The Kruskal-Wallis test was used for nonnormally distributed variables. Pairwise comparisons of groups were made with post hoc tests. The relations between the qualitative variables were compared with Fisher’s exact tests. The significance value was accepted as 0.05 for all statistical analyses. All statistical analyses were performed with the TURCOSA (Turcosa Analytics Ltd Co, Turkey, www.turcosa.com.tr) statistical software program.

## 3. Results

Living conditions were the same for the rats in all groups during the experiment period. There is no death in Group 1 and Group 2 while there was only one death in Group 3 due to aspiration of abemaciclib solution during the feeding via orogastric tube.

### 3.1. Histopathological findings

There were no findings of mucositis in Group 1. On the other hand, there were histological changes mainly in the ileum in Group 2 and Group 3. The histopathological findings mainly consisted of thickening of villus and crypts, mucosal inflammation, edema, and intraepithelial leucocyte accumulation.

**Figure 1:**
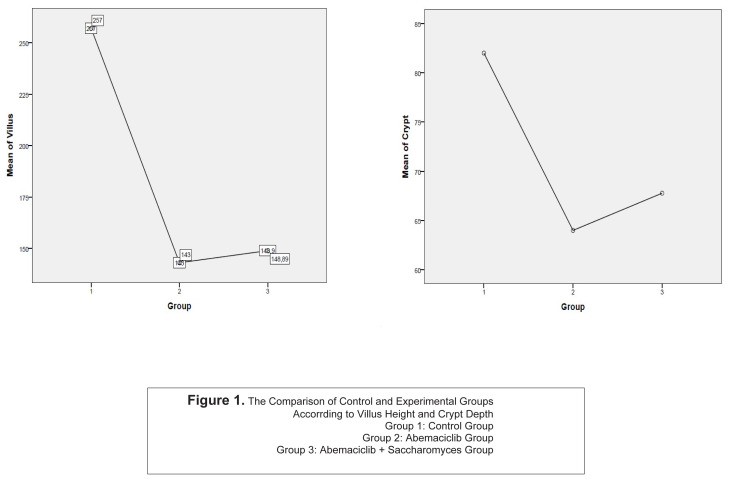
The Comparison of control and experimental groups according to villus height and cryot depth.

**Figure 2:**
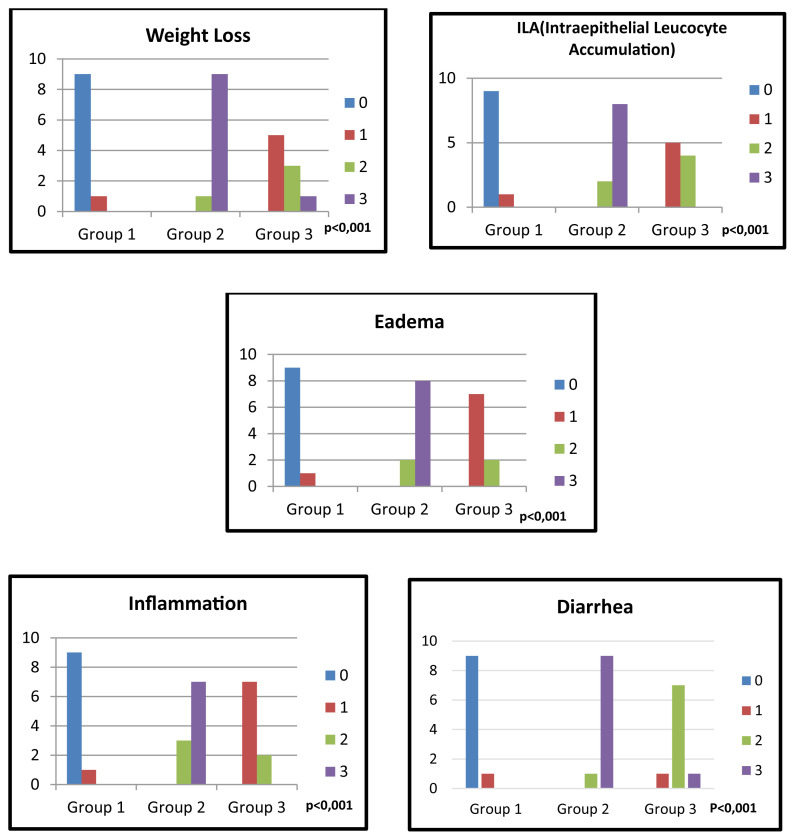
The comparison of control and experimental groups according to severity of weight loss, edema, ILA (intraepithelial leucocyte accumulation), diarrhea and inflammation. Group 1: Control Group, Group 2: Abemaciclib Group, Group 3: Abemaciclib + Saccharomyces Group.

**Figure 3:**
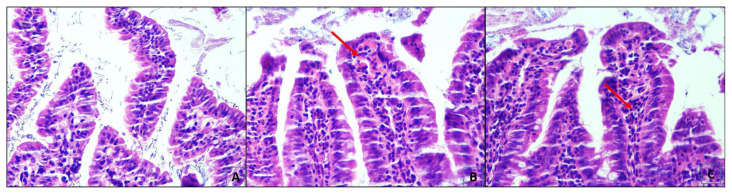
A. Group 1. Intestine of the control group with normal histology, without any inflammation, edema, thickening, orin the mucosal villi (HEX200), B. Group 2. Rats receiving abemaciclib alone showing severe mucosal inflammation (arrow) and thickening in the mucosal villi, and C. Rats receiving abemaciclib and Saccharomyces boulardii showing scant inflammation (arrow) with minimal thickening of the villi (HE X200).

A statistically significant difference was found between Group 1 and Group 2–3 with the comparison of villus heights, crypt depths, and the ratio of these two variables (p < 0.001). But no statistically significant difference was found between Group 2 and Group 3 (Figure 1–3) (p = 0.237).

There was only one rat in Group 1 which shows mild inflammation, mild edema, and mild intraepithelial leucocyte accumulation while the others in Group 1 had no histopathological findings. When we compare Group 2 and Group 3 according to histopathologic findings; there was mild to moderate inflammation, edema, and intraepithelial leucocyte accumulation in Group 3 and moderate to severe findings in Group 2. A statistically significant difference was determined between Group 2 and Group 3 according to comparisons of these histopathological findings (Figure 2) (p < 0.001).

### 3.2. Clinical findings

There was mild diarrhea and weight loss in only one rat in Group 1 while there was no diarrhea and weight loss in the other 9 rats of Group 1. One rat had mild, seven rats had moderate, and one rat had severe diarrhea in Group 3. There was moderate diarrhea in 1 rat and severe diarrhea in 9 rats of Group 2. A statistically significant difference was evaluated between Group 2 and Group 3 in the comparison of the severity of diarrhea (Figure 2) (p < 0.001).

There was mild weight loss in 5 rats, moderate weight loss in 3 rats, and severe weight loss in 1 rat of Group 3. There was moderate weight loss in 1 rat and severe weight loss in 9 rats of Group 2. There was a statistically significant difference between Group 2 and Group 3 in the comparison of weight loss severity (Figure 2) (p < 0.001).

## 4. Discussion

Breast cancer is the most common cancer in women in the World [1]. Especially in the treatment of patients with hormone receptor (HR)-positive and ERBB2 (formerly HER2)-negative advanced breast cancer, CDK4 and CDK6 inhibitors (abemaciclib, ribociclib, and palbociclib) have an important role [15–19]. Diarrhea is the most common gastrointestinal and nonhematological complication with abemaciclib, confirmed by the MONARCH 2 study, while the significant adverse effect of ribociclib and palbociclib is nonfatal neutropenia by suppressing the bone marrow [15, 20]. The management of abemaciclib-induced diarrhea consists of different treatment methods such as antidiarrheal medication (e.g., loperamide), dose adjustments, increase in oral fluids [10].

In the MONARCH 3 study, low-grade diarrhea, which was possible to control this diarrhea with conventional antidiarrheal drugs and dose adjustments, was the most common side effect of the use of abemaciclib [10]. According to the study of Thibault et al., significant intestinal gene expression and stool changes were detected in rats administered abemaciclib alone. Loss of microvilli and terminal mesh were seen; additionally, multiple phagosomes were seen by microscopic examination, which cause vacuolar degeneration of the surface cells of villi. In the same study, abemaciclib causes the proliferation of oligomucous cells, which contain mucin-like cytoplasmic vacuoles, instead of goblet cells, and these cells dominate over the villi and release their secretions into the apical lumen. These changes can be summarised as the proliferation of crypt cells, loss of goblet cells, loss of microvilli, and mucosal inflammation. It was also notable that abemaciclib is the only one of CDK4 and CDK6 inhibitors causing intestinal toxicity in the rats [21].

Especially fluorouracil and irinotecan have been shown with chemotherapeutic induced diarrhea [22]. The toxic effects of fluorouracil depend on the treatment schedule and dose of the drug. Stomatitis and myelosuppression are the main toxic effects of bolus regimens and diarrhea is the main toxic effect of fluorouracil infusion. Irinotecan may cause also dose-limiting diarrhea as a toxic effect independently from the treatment regimens, either bolus or infusion. Tyrosine kinase inhibitors, antimetabolites, monoclonal antibodies, and other chemotherapeutics may cause also complications like enterocolitis, abdominal pain, autoimmune colitis, ischaemic colitis, etc [22]. Abemaciclib, a cyclin-dependent kinase inhibitor is associated with dose-dependent early-onset diarrhea [23]. The mechanisms of chemotherapy-induced diarrhea include; mucosal damage, carbohydrate malabsorption, and fat malabsorption. There are some options for the treatment and prophylaxis of chemotherapy-induced diarrhea; such as glutamine, celecoxib, probiotics, activated charcoal, absorbents, and racecadotril, but there is no evidence of the efficiency of these agents [22]. In our experimental study there was a statistically significant difference between the rats in Group 1 and the rats were administered abemaciclib (Group 2–3) in terms of villus height, crypt depth, edema, inflammation, and intraepithelial leucocyte accumulation. As a result of these histopathological reactions, weight loss progressed and associated with diarrhea in the abemaciclib group.

The first-line treatment option is fluid resuscitation for chemotherapy-induced diarrhea because the patients are generally dehydrated due to diarrhea with or without vomiting. Loperamide treatment is the first option after fluid resuscitation. There is no systemic effect of loperamide treatment, but it can cause paralytic ileus. Codeine may be an alternative to loperamide, but it can cause dose-limiting nausea, flatulence, and sedation. Octreotide, budesonide, atropine, antibiotics, and bile acids sequestrants are the other treatment options for persistent or refractory diarrhea despite loperamide [22].

The use of probiotics has become widespread in recent years as one of these treatment methods. It has been shown that the use of probiotics in several patients with chemotherapy-induced is safe, leads to a decrease in the severity of diarrhea, and prevents body weight loss, therefore it is recommended to be used clinically by cancer patients [24–27]. Since the immune system of patients receiving chemotherapy is severely weakened, it is a crucial point that the use of probiotics may increase the risk of infection and this should be considered when preparing the treatment [28].

The use of *S. boulardii* in the treatment of mucositis has been discussed in recent years. In a meta-analysis by McFarland et al., it was stated that *S. boulardii* both helps prevent antibiotic-associated diarrhea and appears to be beneficial for *Clostridium difficile* infection [14]. D’Souza et al. suggested that probiotics, especially *S. boulardii* and lactobacilli, can be used to prevent antibiotic-associated diarrhea, but the efficacy of probiotics in the treatment of antibiotic-associated diarrhea has not been proven [29]. Another trial using probiotics as preventative agents should consider the costs and need for routine use of these agents [29]. On the contrary, Maioli et al. studied that *S. boulardii* could not prevent the effects of 5-Fluorouracil-induced experimental mucositis [30]. Among the treatment methods for diarrhea due to mucositis induced by irinotecan, there is also the use of probiotics containing *S. boulardii*. In the study of Sezer et al., it was found that *S. boulardii* provided significant improvement in diarrhea and mucositis caused by irinotecan [31]. In our experimental study, there was a statistically significant difference between Group 2 and Group 3 in terms of edema, inflammation, and intraepithelial leucocyte accumulation, but, when the villus height and crypt depth of the two groups were examined, there was no statistically significant difference. However, there was a statistically significant decrease in weight loss and diarrhea in Group 3 in comparison to Group 2 due to the administration of *S. boulardii* in Group 3.

Loperamide is used in the management of chemotherapy-induced diarrhea since it was used in the MONARCH 2 study, while there is no proven positive effect yet [16]. Besides, in the nextMONARCH study; abemaciclib-induced diarrhea is dose-dependent and early use of antidiarrheal therapy is enough for the management without avoidance of dose reductions or interruptions of abemaciclib therapy. Constipation was seen in the prophylactic loperamide treatment group [23].

Considering that Saccharomyces is a low-cost, easy-to-use, nontoxic, and nontoxic probiotic, it may be an effective treatment method in patients who develop diarrhea due to abemaciclib. However, this idea needs to be supported by experimental studies on humans. The fact that our study was an experimental study on rats, the need for human studies, the number of rats, and the fact that it was not compared with another treatment method can be considered as limitations.

## 5. Conclusion

Our study aims to investigate the protective effect of a probiotic agent, *S. boulardii*, which can be effective in diarrhea associated with malignancy treatment, CDK4 and CDK6 inhibitors are used in this study. To conclude, *S. boulardii* should be considered as a treatment option for chemotherapy-induced diarrhea. As there is no other comparison study, further comparative studies with the other treatment options and in vivo human randomized controlled studies can be conducted in the future.
